# Revolution at the Corner Drugstore

**DOI:** 10.6004/jadpro.2016.7.3.2

**Published:** 2016-04-01

**Authors:** Pamela Hallquist Viale, Matthew Farber,(1), Patricia J. Goldsmith,(2), Kate D. Jeffers,(3), Wendy J. Smith,(4)

**Affiliations:** (1) Oncology Disease State Management at Walgreens; (2) CancerCare; (3) UCHealth–Memorial Hospital, Colorado Springs, Colorado; (4) Meniscus Educational Institute, Lawrenceville, New Jersey

An expanding armamentarium of oral oncologic drugs represents a revolution in the treatment of cancer but also presents significant challenges to adherence and the management of costs, side effects, and a growing clinician workload.

Most often, identifying the urgent needs of cancer patients, including financial support and education, falls to the health care giver, whichincludes advanced practitioners, put forth participants in a panel discussion at JADPRO Live at APSHO. Panelists agreed that the search for resources needs to be comprehensive and should involve specialty pharmacy, community pharmacy, foundations, and even social workers.

Panel moderator Pamela Hallquist Viale, RN, MS, CNS, ANP, set the stage for the discussion by noting the sea change in the treatment of cancer, namely, a transition from mostly or all-intravenous medications to oral agents. The current crop of oral medications is just the tip of the iceberg, as a large proportion of oncology drugs in the pipeline are orals.

Although oral medications are easier for patients to take, they can be costly, and unlike infusions, no nurse is present for administration. This creates opportunities for noncompliance and nonadherence, said Ms. Viale.

The expense attached to oral drugs has not only affected adherence but also nurse workload, as these agents often require preauthorization and the need to connect patients with funding sources if they cannot afford the drugs, said Wendy Smith, RN, MSN, ACNP, AOCN®, Director of Interprofessional Education, Meniscus Educational Institute.

At her previous institution, processes were established in 2012 to identify staff that would be responsible for patient education once a prescription is written for an oral drug. "Doctors were writing prescriptions for oral medications and patients were not getting the teaching for chemotherapy because the nurses didn’t even know they got the prescriptions," she said.

"I’m a proponent of advanced practitioners. We can take the leadership, being creative and coming up with innovative processes," Ms. Smith commented. For those who need help developing these processes, she advised visiting the Association of Community Cancer Centers website and searching for its algorithm.

To keep drug protocols up to date and to assist with preventing drug-drug interactions and other issues related to polypharmacy, Ms. Smith wrote a proposal to employ a pharmacist as a provider on her team.

## OVERCOMING BARRIERS TO TREATMENT

Monitoring adherence is a critical component of successful treatment, added Kate D. Jeffers, PharmD, BCOP, ambulatory oncology clinical pharmacy specialist at UCHealth–Memorial Hospital, Colorado Springs, Colorado.

"After an initial prescription, we call the patient within 7 days to make sure that they received their medication and are actually taking it," Dr. Jeffers said. Adherence is monitored at every clinical encounter. Clinicians are aware before each visit whether the patient is adhering to treatment, and if they aren’t, what the barriers to adherence might be. This practice "allows our providers to better target their visits," she pointed out.

About half of cancer patients who are not adherent to their drug regimens cite cost as the reason, said Ms. Viale. The pharmacy at her county hospital hired a technician to manage the patient assistance program, which saved the hospital $3 million in the first year. "It does pay to hire someone to handle some of the intricacies of the program," she noted.

As an extension of the care community, Walgreens has, as its mantra, been proactive in seeking financial assistance, said Matthew Farber, MA, the Senior Director of Oncology Disease State Management at Walgreens. "We want to help you help your patients get access to care," he said. "This is what I’m training my staff to do."

Handling oncology prescriptions is already a complicated process that will become even more so as more targeted therapies and oral therapies enter the market, he indicated. Of the approximate 800 oncology drugs in development, about 40% are oral medications, and of these, 80% are first in class. "That means that a lot of education will need to take place," said Mr. Farber.

The CancerCare Foundation, based in New York City, offers support services and financial assistance to patients with cancer. Sixty percent of individuals who call CancerCare are reaching out for financial assistance, said Patricia J. Goldsmith, chief executive officer at CancerCare.

An inordinate amount of cost shifting means that some oral medications now carry a 50% co-pay. The CancerCare Co-Payment Assistance Foundation is authorized by the Office of the Inspector General to establish co-pay assistance funds for patients with cancer. "In some circumstances, that can be as high as $10,000 per year," she said.

Financial support goes beyond co-pay subsidies and includes assistance for transportation needs. "We distributed over $5 million for transportation assistance last year," said Ms. Goldsmith.

Higher out-of-pocket costs translate to lower rates of medication persistence, she added. Ms. Smith noted that as many as three of four patients stop their drugs within 1 year, "and it’s increasing our health-care costs overall...It’s just a vicious cycle."

Some cancer patients are receiving split fill prescriptions (every 2 weeks instead of monthly) because of this high rate of nonadherence, she said. Educating patients about the drugs they’re taking, their importance, and the correct schedule is therefore paramount to combatting nonadherence and to reducing the amount of avoidable health-care costs.

"We cannot afford to be bystanders," Ms. Smith emphasized. "I feel that nurse practitioners, physician assistants, and pharmacists need to be on the board of everything. We need to be advocates."

## A BURGEONING WORKLOAD

The move toward oral oncologic drugs has already resulted in a shift of responsibilities to clinic nurses in educating patients, monitoring adherence, and finding patient assistance programs. Their work will become even more time consuming as the number of oral oncologic agents increases, speakers predicted.

With limited distribution of some oral oncologic drugs and restrictions on where prescriptions can be filled, depending on the insurer, clinicians may be unaware when patients’ prescriptions go unfilled. "Adherence is a constant effort," said Mr. Farber. "If you send it to us and we can’t fill it, we will triage it to someone who can and we will communicate that back to the provider, to ensure that they are kept in the loop."

Dr. Jeffers utilizes specialty pharmacy as much as possible to ease the burden on nurses. "Assistance with adherence and financial programs is exactly what the field needs. And this is the only way our practices are going to be able to survive and still see patients, and not be stuck on the phone the entire time," she said.

"At CancerCare, our Co-Pay Foundation works very closely with all of the manufacturers to understand their programs," added Ms. Goldsmith. "If a manufacturer has a program, we refer the patient directly to the manufacturer, and it’s somewhat of a ’warm’ transfer."

Developing relationships with neighborhood pharmacists or specialty pharmacists is an invaluable tool for recognizing the potential for drug-drug interactions, added Ms. Smith, including interactions with over-the-counter medicines and herbals.

